# Taxonomic and Functional Diversity of Soil and Hypolithic Microbial Communities in Miers Valley, McMurdo Dry Valleys, Antarctica

**DOI:** 10.3389/fmicb.2016.01642

**Published:** 2016-10-20

**Authors:** Sean T. S. Wei, Donnabella C. Lacap-Bugler, Maggie C. Y. Lau, Tancredi Caruso, Subramanya Rao, Asunción de los Rios, Stephen K. Archer, Jill M. Y. Chiu, Colleen Higgins, Joy D. Van Nostrand, Jizhong Zhou, David W. Hopkins, Stephen B. Pointing

**Affiliations:** ^1^Institute for Applied Ecology New Zealand, School of Applied Sciences, Auckland University of TechnologyAuckland, New Zealand; ^2^Department of Geosciences, Princeton UniversityPrinceton, NJ, USA; ^3^School of Biological Sciences, Queen’s University BelfastBelfast, Northern Ireland; ^4^Department of Health Technology and Informatics, Hong Kong Polytechnic UniversityHong Kong, China; ^5^Departamento de Biogeoquímica y Ecología Microbiana, Museo Nacional de Ciencias NaturalesMadrid, Spain; ^6^Department of Biology, Hong Kong Baptist UniversityHong Kong, China; ^7^Department of Microbiology and Plant Biology, Institute for Environmental Genomics, University of OklahomaNorman, OK, USA; ^8^State Key Joint Laboratory of Environment Simulation and Pollution Control, School of Environment, Tsinghua UniversityBeijing, China; ^9^Earth Sciences Division, Lawrence Berkeley National LaboratoryBerkeley, CA, USA; ^10^School of Agriculture, Food and Environment, The Royal Agricultural UniversityGloucestershire, UK; ^11^Institute of Nature and Environmental Technology, Kanazawa UniversityKanazawa, Japan

**Keywords:** Antarctica, cyanobacteria, Dry Valleys, Geochip, hypolith, soil

## Abstract

The McMurdo Dry Valleys of Antarctica are an extreme polar desert. Mineral soils support subsurface microbial communities and translucent rocks support development of hypolithic communities on ventral surfaces in soil contact. Despite significant research attention, relatively little is known about taxonomic and functional diversity or their inter-relationships. Here we report a combined diversity and functional interrogation for soil and hypoliths of the Miers Valley in the McMurdo Dry Valleys of Antarctica. The study employed 16S rRNA fingerprinting and high throughput sequencing combined with the GeoChip functional microarray. The soil community was revealed as a highly diverse reservoir of bacterial diversity dominated by actinobacteria. Hypolithic communities were less diverse and dominated by cyanobacteria. Major differences in putative functionality were that soil communities displayed greater diversity in stress tolerance and recalcitrant substrate utilization pathways, whilst hypolithic communities supported greater diversity of nutrient limitation adaptation pathways. A relatively high level of functional redundancy in both soil and hypoliths may indicate adaptation of these communities to fluctuating environmental conditions.

## Introduction

The largest ice-free region in Antarctica is the McMurdo Dry Valleys (hereafter referred to as Dry Valleys) and these are designated by international treaty as an Antarctic Special Managed Area to reflect their unique ecological status (SCAR, 2004). The environment is characterized by extremely low temperatures, moisture deficit, oligotrophic soils, and a harsh irradiance regime (Wynn-Williams, 1990). Concentrations of biological productivity occur at lakes, but these are scarce and occupy a small fraction of the Dry Valleys landscape (Taton et al., 2006; Hopkins et al., 2009). Conversely the valleys support expansive mineral soils and rocky outcrops and these support the largest standing biomass in the Dry Valleys (Cowan and Tow, 2004; Cary et al., 2010; Cowan et al., 2014). Here communities develop in cryptic niches that allow avoidance of environmental extremes (Pointing and Belnap, 2012). The Dry Valleys soil has been demonstrated as microbiologically distinct from all other soils worldwide in a metagenomic study (Fierer et al., 2012). Key differences were the significantly reduced biodiversity in Antarctic soils, relatively high abundance of stress and dormancy related genes and the reduced abundance of antibiotic resistance genes that implied less interspecific competition in these soils.

A major focus of recent research has been elucidating the biodiversity of ice-free Dry Valleys landscapes. In all edaphic substrates examined to date the archaea have been shown to be absent or a minor component and communities largely comprise bacteria and eukaryotic microorganisms (Pointing et al., 2009). Soils are highly oligotrophic and support relatively low biomass (Pointing et al., 2009). Surface soil biota comprises diverse bacterial phyla, with heterogeneous communities dominated by the Actinobacteria (Pointing et al., 2009; Lee et al., 2012). Eukaryotic soil assemblages are largely fungal and dominated by few ascomycete taxa (Pointing et al., 2009; Rao et al., 2011). Recent studies suggest there may also be appreciable phage diversity in Antarctic soils and hypoliths (Zablocki et al., 2014; Wei et al., 2015). Major drivers of differences in soil diversity appear to be organic carbon and soluble salts in soils (Pointing et al., 2009; Magalhães et al., 2012). This surface layer comprises the “critical zone” of biological activity in dryland landscapes (Pointing and Belnap, 2012), because a dramatic decline in biodiversity and activity occurs in subsurface layers (Stomeo et al., 2012). Particular attention has also been directed toward the hypolithic habitat in desert pavement terrain (Chan et al., 2012; Pointing et al., 2015; Pointing, 2016), since Dry Valleys soils also support extensive desert pavement substrates with quartz, marble and other translucent stones embedded in the soil surface (Wynn-Williams, 1990). The ventral surfaces of stones provide a degree of buffering from the harsh abiotic stressors and they support “islands” of relatively high biomass amid the depauperate soil surroundings (Pointing et al., 2009; Cowan et al., 2010). Several studies have revealed communities that develop on these stones are dominated by cyanobacteria and assemble under different abiotic and biotic interactions compared to soil communities including moisture, UV and thermal regime as well as interspecific interactions (Wood et al., 2008; Pointing et al., 2009; Cowan et al., 2010; Makhalanyane et al., 2013; de los Rios et al., 2014).

A major barrier to further understanding this intriguing dual-niche habitat is the lack of data on functional aspects for the hypolith and soil communities (Cowan et al., 2014). We regard this as important not only to identify the spatial distribution of productivity and catabolism, but also to assist in predicting resilience to change in the system. A limited number of *in situ* respirometry studies have implicated microbial activity in carbon and nitrogen transformations for the hypolith-soil system (Cockell and Stokes, 2004; Hopkins et al., 2006, 2009; Cowan et al., 2011). Despite this, almost nothing is known about the contribution of these communities toward metabolic processes essential to biological mineral transformation, or the stress tolerance mechanisms that facilitate their survival under extreme environmental stress. We recently reported the first study of functional diversity for cryptic communities in a rocky, high altitude, inland Dry Valley location using the Geochip functional microarray, and identified that the ability to transform recalcitrant aromatics explained the major difference between rock and soil communities (Chan et al., 2013). However, a knowledge gap remains for the maritime dry valleys ecosystem, where major impacts of environmental change are predicted (Doran et al., 2002), but information on functionality of the ecosystem is scarce.

We tested the hypothesis that communities in soil and hypolithic niches within the Miers Valley (McMurdo Dry Valleys, Antarctica) show divergence in both taxonomic composition and putative functional traits. The Miers Valley is a maritime-influenced Dry Valley with expansive moraine valley floor that presents a hypolith-soil habitat. The approach employed high throughput pyrosequencing combined with metagenomic interrogation using the Geochip functional microarray, to address key issues relating to microbial functional ecology in a long-term terrestrial Antarctic study site.

## Materials and Methods

### Field Location, Microclimate, and Biological Sampling

Miers Valley occupies a maritime location within the McMurdo Dry Valleys Antarctic Special Managed Area. The valley lies between the latitudes 78°060′ S and 78°070′ S and longitudes 163°440′ E and 164°120′ E and comprises a wide valley floor characterized by hypolith-soil terrain and moraine deposition. A field survey was conducted in January 2011 at a hypolith-soil location in the central part of the valley (78°05.486′ S, 163°48.539′ E). Microclimate variables at the location were previously reported for this long term study site: mean moisture content of 0.53%, conductivity = 0.3 μS, C and N both <1% w/w, and C:N = 18.2 (Lee et al., 2012). The natural frost polygon topography was used to identify ten sampling locations (one polygon = one location), and at each location a hypolith from near the polygon center was sampled (*n* = 10). Hypolith-soil interface soil was sampled (*n* = 10), hypolith subsurface soil at 50 mm depth (*n* = 10) and hypolith subsurface soil at 100 mm depth (*n* = 10). In addition a ‘control’ bare soil site without hypolith at least 1 m distant from any hypolith was sampled the same way (total *n* = 30). The 70 samples were retrieved as described previously (Pointing et al., 2009). Briefly, for each sample 50 g soil was introduced aseptically to sterile sample containers after removing the surface grains of mobile wind-blown sand; hypolith biomass (standardized at 2 cm^2^) was scraped aseptically from stones and introduced to sterile sample containers. All samples were stored frozen in the field in darkness, and subsequently at -80°C in the laboratory until processed.

### DNA Recovery and t-RFLP Community Profiling

Environmental DNA recovery was achieved separately for each sample (10 g soil or 2 cm^2^ hypolith) by lysis in CTAB with lysozyme and RNAse, followed by phenol:chloroform extraction at 60°C (Pointing et al., 2009). Soil samples were gently mixed by shaking for 10 s prior to DNA extraction. Recovered DNA was quantified using Nanodrop (Thermo-scientific) and template for all samples normalized at 100 ng/DNA per reaction. The PCR reaction comprised a 25 μl PCR mixture containing 0.1–2 μl of DNA template, 0.5 μM of each primer, 2.5 units of high fidelity *Taq* polymerase (Takara, Beijing, China^1^), 1x PCR buffer provided by the manufacturer, 200 μM of each dNTP, and ddH_2_O. Amplification of 16S rRNA genes was achieved using primer pair 341F-CCTACGGGAGGCAGCAG and 907R-CCGTCAATTCMTTTGAGTTT (Muyzer et al., 1993). PCR reactions for t-RFLP analysis were carried out using a FAM-labeled forward primer as previously described (Pointing et al., 2009). The PCR reaction involved an initial denaturation time of 5 min; 30 cycles at 95°C for 1 min, 55°C for 1 min, 72°C for 1 min, and a final extension at 72°C for 10 min. Positive and negative controls were run for every PCR. Gel-purified amplicons were digested using three restriction enzymes *Hae* III, *Hinf* 1, *Msp* 1 (ThermoFisher, Hong Kong, China^2^) and the most informative selected for further analysis (*Msp* 1). Fragment analysis was achieved by capillary electrophoresis (Applied Biosystems 3730 Genetic Analyzer), using a GeneScan ROX-labeled GS500 internal size standard from the manufacturer. A total of eight samples were excluded from analysis due to lack of PCR amplification, thus 62 samples were subjected to further analysis. The t-RFLP patterns and quality were analyzed using the freeware PeakScanner^TM^ (version 1.0) (Applied Biosystems^3^) and a data matrix comprising fragment size and abundance was generated. The software Perl and R^4^ were then used to identify true peaks from artifacts among the terminal restriction fragment sequences and bin fragments of similar size as previously described (Abdo et al., 2006). Peaks within three standard deviations of the baseline noise signal were excluded. The relative abundance of a terminal restriction fragment within a given t-RFLP pattern was generated as a ratio of the respective peak area to total area of all peaks. A virtual digest using *Msp* I was carried out on sequences from our extensive curated 16S rRNA library for Antarctic bacteria (Pointing et al., 2009) and this allowed assignment of phylogenetic identity to over 90% of t-RFLP peaks. Those peaks within 1bp of another were regarded as representing the same taxon.

### Pyrosequencing and Phylogenetic Assignment

The two most representative samples for each statistically supported grouping as delineated in the t-RFLP analysis were further interrogated via barcoded pyrosequencing using the Roche GS Junior System (454 Life Sciences Corp., Branford, CT, USA). Since the objective was to estimate diversity in samples, we delineated the most representative samples on the basis of greatest number of shared operational taxonomic units (OTUs) as indicated by t-RFLP analysis. Amplification of 16S rRNA genes was achieved using primer pair 341F and 907R (Muyzer et al., 1993) with PCR conditions as described above. For each amplicon library (*n* = 2) purification was carried out with Agencourt AMPure XP Bead (Beckman Coulter, CA, USA^5^) according to manufacturers instructions. The library was quantified with Quant-iT PicoGreen dsDNA Assay Kit (Invitrogen Life Technologies, NY, USA^2^) using FLUOstar OPTIMA F fluorometer (BMG Labtech GmbH, Offenburg, Germany^6^) and library quality was assessed with the FlashGel System (Lonza Group Ltd., Basel, Switzerland). Emulsion-PCR was carried out with GS Junior Titanium emPCR Kit (Lib-L, 454 Life Sciences Corp., CT, USA^7^) according to the emPCR Amplification Method Manual – Lib-L, Single-Prep. The sequencing reaction was carried out with the GS Junior Titanium Sequencing Kit and GS Junior Titanium PicoTiterPlate Kit (454 Life Sciences Corp.) according to the manufacturers instructions. The sequencing run was conducted in 200 cycles.

Pyrosequencing reads were sorted according to barcoding prior to analysis and processing using the software package MOTHUR (Schloss and Handelsman, 2005). De-noising was carried out with sequences removed from analysis if they met any of the following criteria: the length was shorter than 300 bp; with an average quality score less than 25; contained ambiguous characters or more than six homopolyers; or did not contain the primer sequence or barcode. In order to remove sequences that were probably due to pyrosequencing errors, sequences were pre-clusted using a pseudo-single linkage algorithm as implemented in MOTHUR. Chimera check was performed using UCHIME with the *de novo* mechanism (Edgar et al., 2011). Hierarchical clustering was performed with the remaining sequences to form clumps that were small enough to align using USEARCH (Edgar, 2010). A master set was created using the longest sequence from each clump. Sequences in the clumps and master set were aligned using MUSCLE (Edgar, 2004). The aligned sequences were merged into a final alignment with the master set as a guide. Alignment columns containing more than 90% gaps were trimmed using trimAL (Capella-Gutiérrez et al., 2009). To correct the differences in sequencing depth among individual samples, the datasets were rarefied to 2,600 sequences. Rarefaction was carried out using MOTHUR (Schloss and Handelsman, 2005) and phylogenetic trees constructed with FastTree (Price et al., 2009) to compare phylogenetic similarity between samples as calculated by the weighted UniFrac metrics (Lozupone et al., 2007). The distances in UniFrac matrix were calculated based on the fraction of branch length shared between two communities within a phylogenetic tree. Alpha diversity was assessed by constructing the rarefaction curves defined at 97% sequence similarity cutoff for OTUs. Taxonomic classification of 16S rRNA gene sequences was made using the ribosomal database project Classifier (Wang et al., 2007). Sequence data have been deposited in NCBI’s sequence read archive under accession number SRA052054.1.

### GeoChip Functional Microarray Analysis

Functional gene diversity was assessed in triplicate for soil and hypolith samples using the GeoChip microarray (He et al., 2007). The GeoChip microarray primarily targets bacterial genes, but a number of archaeal and fungal genes are also represented by oligonucleotides in the array. Consequently, our GeoChip analysis considered all three domains GeoChip 4 contains 84,000 50-mer oligonucleotide probes covering 152,000 gene variants (i.e., individual sequences from a gene) from 401 distinct functional gene categories involved in major biogeochemical, ecological, and other processes. (12 categories: biogeochemical cycling of carbon, nitrogen, phosphorus and sulfur; resistance to metal and antibiotics; energy process; organic compound remediation; stress response; bacteriophage-related; virulence-related and others) (Tu et al., 2014). Hybridization was carried out from triplicate arrays as previously described (Chan et al., 2013). The normalized hybridization output data were then re-organized based upon functional category. Output from the array data was grouped into functional categories related to major metabolic processes. The level of redundancy in the large number of pathway-specific GeoChip oligonucleotides, allowed a high degree of confidence in signal recovery inferring occurrence of any given pathway. Among these probes, 34,464 probes were derived from genes involved in carbon, nitrogen cycling, and stress responses. Hybridization of DNA from hypolith and soil samples was achieved with an average of 50.6% of the probes, covering 88.6% of the targeted genes of interest on GeoChip 4. The GeoChip dataset is publicly available at http://ieg.ou.edu/4download.

### Statistical Analyses

Statistical analysis on t-RFLP-derived community profiles was conducted as follows: Alpha diversity indices (Shannon’s Index [H’], Simpsons Diversity Index [D], Pielou’s Evenness [J’]) were calculated using untransformed data. Differences stated as significant were tested using one-way and two-way analysis of variance (ANOVA), or analysis of similarity (ANOSIM). The ANOSIM produces a statistic R which is based on the difference of mean ranks between groups and within groups. A value approaching to 1 indicates the assemblage composition is totally different where a value of 0 indicates no difference. Multivariate analysis of diversity data was performed on square-root transformed diversity data. Bray–Curtis Similarities and de-trended correspondence analysis (DCA) were visualized in two-dimensional plots. The Bray–Curtis index was calculated for each sample and their dissimilarity reflected in a two-dimensional plot where greater spatial separation correlated to greater dissimilarity. Significance testing was achieved using ANOSIM. The DCA approach was used to illustrate gradients in species-rich but sparse diversity matrices as commonly encountered in Antarctic soils. Here the two-dimensional plot shows that taxa with greater distance from others explain more of the observed differences in diversity between the two substrates. No significance testing is possible with DCA. Differences between soil and hypolith functional gene populations were tested using ANOVA and expressed as an *F* statistic with significance testing. All analyses were performed using Primer v6.1.6 (Clarke, 1993). All results stated as significant have a confidence level of *P* < 0.05 unless stated otherwise.

We used a Mantel test to test the hypothesis that communities that diverge in structure (t-RFLP-defined diversity) also diverge in putative functional traits (GeoChip data). For this test, raw data were scaled to zero mean and unit variance to calculate a matrix of *z*-scores (Gotelli and Ellison, 2004), from which Euclidean distance between samples were then obtained. The resultant functional and community dissimilarity matrices were then correlated with the Mantel test (Legendre and Legendre, 1998). For example, with this test we could verify the hypothesis that increased dissimilarity in taxonomic composition correlates with increased dissimilarity in the functions expressed by the communities.

## Results

### Taxonomic Diversity

**FIGURE 1:**
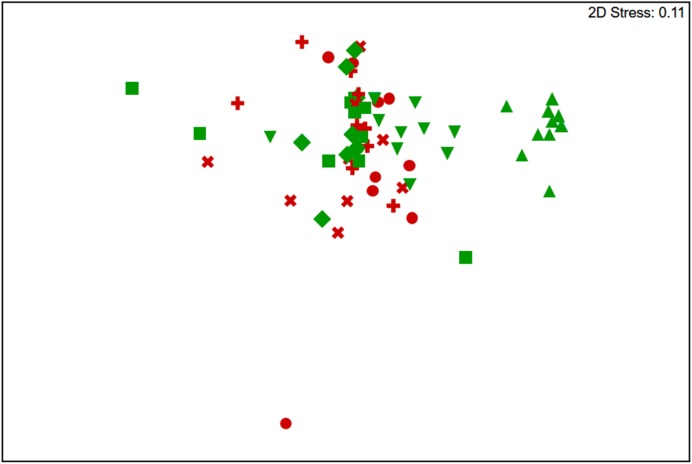
**Non-metric multidimensional scaling plot of Bray–Curtis similarities for t-RFLP-defined bacterial communities recovered from hypolith (green triangles), hypolith-soil interface (inverted green triangles), soil 50 mm below hypolith (green squares), soil 100 mm below hypolith (green diamonds), bare surface soil (red circles), soil 50 mm below bare soil (red +), soil 100 mm below bare soil (red x).** No difference was observed between soil samples regardless of location (ANOSIM *R* = 0.006, *p* = 0.305), whilst soils and hypoliths were significantly different (ANOSIM *R* = 0.789, *p* = 0.001).

The t-RFLP analysis confirmed that hypolith and soil supported significantly different communities [ANOSIM Global *R* = 0.789, *p* = 0.001 (permutation = 999, *n* = 40)] (**Figure 1**). However, there was no major difference in diversity between soil at the hypolith-soil interface or below hypoliths, or surface and subsurface soils 1 m away from hypoliths (**Figure 1**). Alpha diversity metrics supported the identification of soils as more diverse and with greater evenness (Soil *H*′ = 8.1, *D* = 0.99, *J*′ = 1.01) compared to hypoliths (Hypolith *H*′ = 5.8, *D* = 0.94, *J*′ = 0.83). Assignment of phylogenetic identity and binning t-RFLP fragments into autotrophs (Cyanobacteria and chemoautotrophs) and heterotrophs (all other bacteria) allowed calculation of the producer/consumer ratio (P/C) which was significantly higher for hypoliths (P/C = 3.28 ± 0.8, *n* = 10) than soils beneath hypoliths (P/C = 1.04 ± 0.39, *n* = 27) or open soils (P/C = 1.23 ± 0.37, *n* = 28). A de-trended correspondence analysis revealed that the Acidobacteria, Cyanobacteria and unidentified bacterial phyla accounted for the greatest differences between communities (**Figure 1**; Supplementary Figure S1).

**FIGURE 2:**
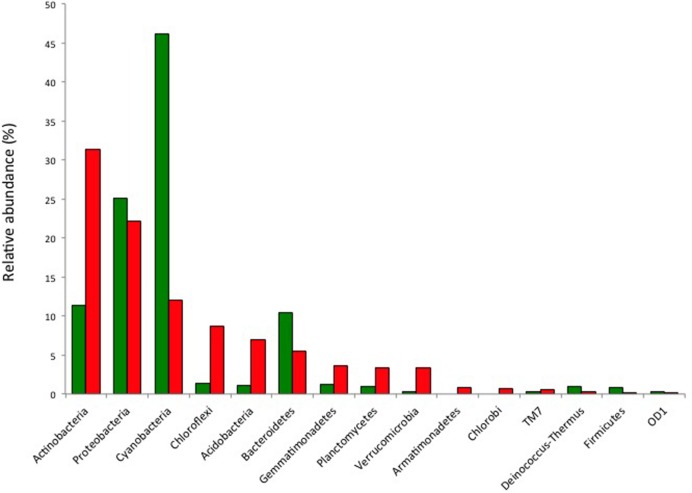
**Relative abundance of bacterial phyla in hypolith (green) and soil (red) communities as determined by pyrosequencing of 16S rRNA genes.** Other phyla present in soils at low abundance and not shown in the bar chart are: Elusimicrobia, Nitrospirae, OP3, WS3, WS5 and three unidentified phyla.

High throughput sequencing was used to further elucidate the diversity of soil and hypoliths. This revealed the phylogenetic basis for the hypolith-soil community differences and validated the t-RFLP community screening data in that the dominant phyla recovered were the same using both approaches (Supplementary Figure S2), although high-throughput sequencing revealed far greater overall diversity than the t-RFLP approach. Soils supported greater phylum level and genus level diversity than hypoliths (**Figure 2**; Supplementary Figure S2). Among the 800 soil OTUs, and 220 hypolith OTUs there were 83 shared OTUs. The most abundant phylum in hypoliths was the Cyanobacteria (46%), whereas in soils, Actinobacteria (31%) were most abundant. The Proteobacteria and Bacteroidetes were the only other phyla to comprise >10% of the overall community, although overall diversity spanned 23 phyla. Identification of cyanobacterial OTUs using RDP Classifier (Wang et al., 2007) revealed the most abundant was a *Synechococcus*-like taxon, whilst all other phylotypes showed highest similarity with oscillatorian genera including *Microcoleus*, *Phormidium*, and *Oscillatoria*. There were no recoverable phylotypes from the nitrogen-fixing family Nostocales. An interesting observation was that while several closely related oscillatorian phylotypes (OTUs 262, 348, 777, 932) were far more common in hypoliths than soil, a single oscillatorian phylotype (OTU 122) displayed almost threefold greater abundance in soil.

### Taxonomic vs. Functional Diversity

**FIGURE 3:**
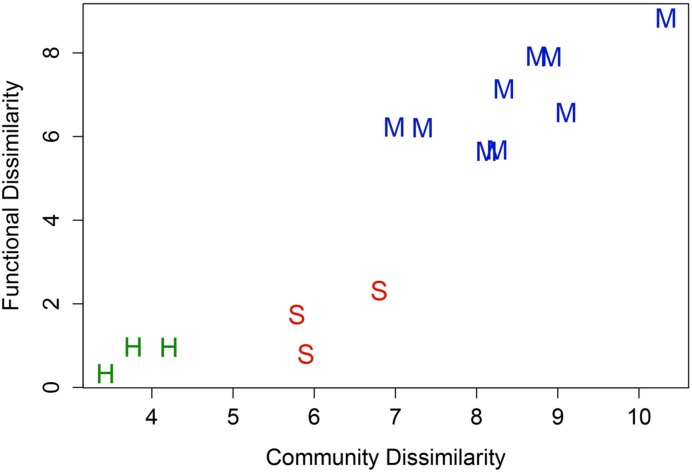
**Relationship between taxonomic (t-RFLP) and functional (GeoChip) diversity for soil and hypolith communities and in pairwise comparisons (*R* = 0.92, *P* < 0.05).** Hypolith, green symbols; Soil, red symbols; pairwise comparisons, blue symbols. The linearity of the plot indicates both communities displayed a similar diversity-functionality relationship, although the greater spatial turnover in soils resulted in overall higher beta diversity for soil communities.

The relationship between 16S rRNA t-RFLP-defined taxonomic diversity and Geochip-defined functional diversity for soil and hypolith communities is shown in **Figure 3**. For the bacteria, functional and taxonomic dissimilarity increased linearly because between-habitat dissimilarity was greater than within-habitat dissimilarity. The linearity of the plot (*R*^2^ = 0.989) suggests that both hypoliths and soil communities displayed a similar diversity-functionality relationship. The GeoChip microarray was able to identify functional genes for archaea and fungi as well as bacteria, and so data for all three domains are reported below for carbon and nitrogen transformation plus stress responses.

### Carbon Transformation Pathways

**FIGURE 4:**
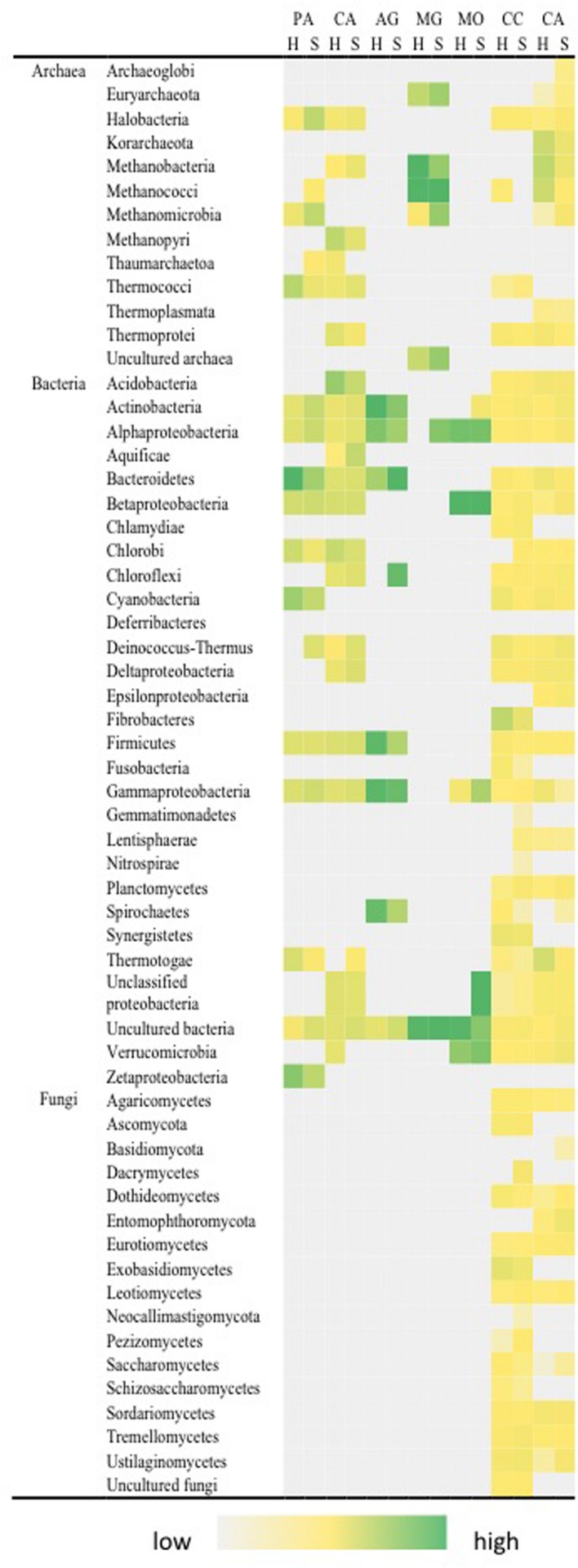
**Distribution of pathways for carbon transformation in soil (S) and hypolith (H) communities.** PA, photoautotrophy; CA, chemoautotrophy; AG, acetogenesis; MG, methanogenesis; MO, methane oxidation; CC, carbohydrate catabolism; CA, aromatic compound catabolism. Color shading indicates relative hybridization intensity.

Carbon fixation was indicated by photoautotrophic and chemoautotrophic pathways for both hypolith and soil communities (**Figure 4**). We included all gene variants that are known to encode enzymes critical to both modes of carbon fixation, although some variants may perform alternative roles and, therefore, some overestimate in this category may have occurred. Of interest was the taxonomic breadth of taxa involved, spanning 12 bacterial and 8 archaeal phyla. Acetogenesis and methane oxidation appeared to be exclusively bacterial transformations: Proteobacteria, Actinobacteria, Bacteroidetes, Firmicutes, and Chloroflexi; Proteobacteria and Verrucomicrobia, respectively, whilst methanogenesis was exclusively an archaeal pathway (Methanococci and Methanobacteria) although some low levels of false positive signal for bacterial phyla was also apparent. The ability to transform carbohydrate substrates was widespread among all archaeal, bacterial and fungal phyla, and surprisingly so was the ability to transform recalcitrant aromatic compounds. There were significant differences in autotrophic pathways. Hypoliths supported a significantly greater signal for cyanobacterial photoautotrophy (*F* = 19.461, *P* < 0.01) after excluding non-cyanobacterial Rubisco variants, although soils supported an overall greater carbon fixation signal than hypoliths (*F* = 17.197, *P* < 0.05). Hypoliths displayed significantly greater signal for the C1 carbon pathways for acetogenesis (*F* = 23.467, *P* < 0.01). Conversely, soils displayed significantly greater signal for aromatic compound transformation (*F* = 23.076, *P* < 0.01).

### Nitrogen Transformation Pathways

**FIGURE 5:**
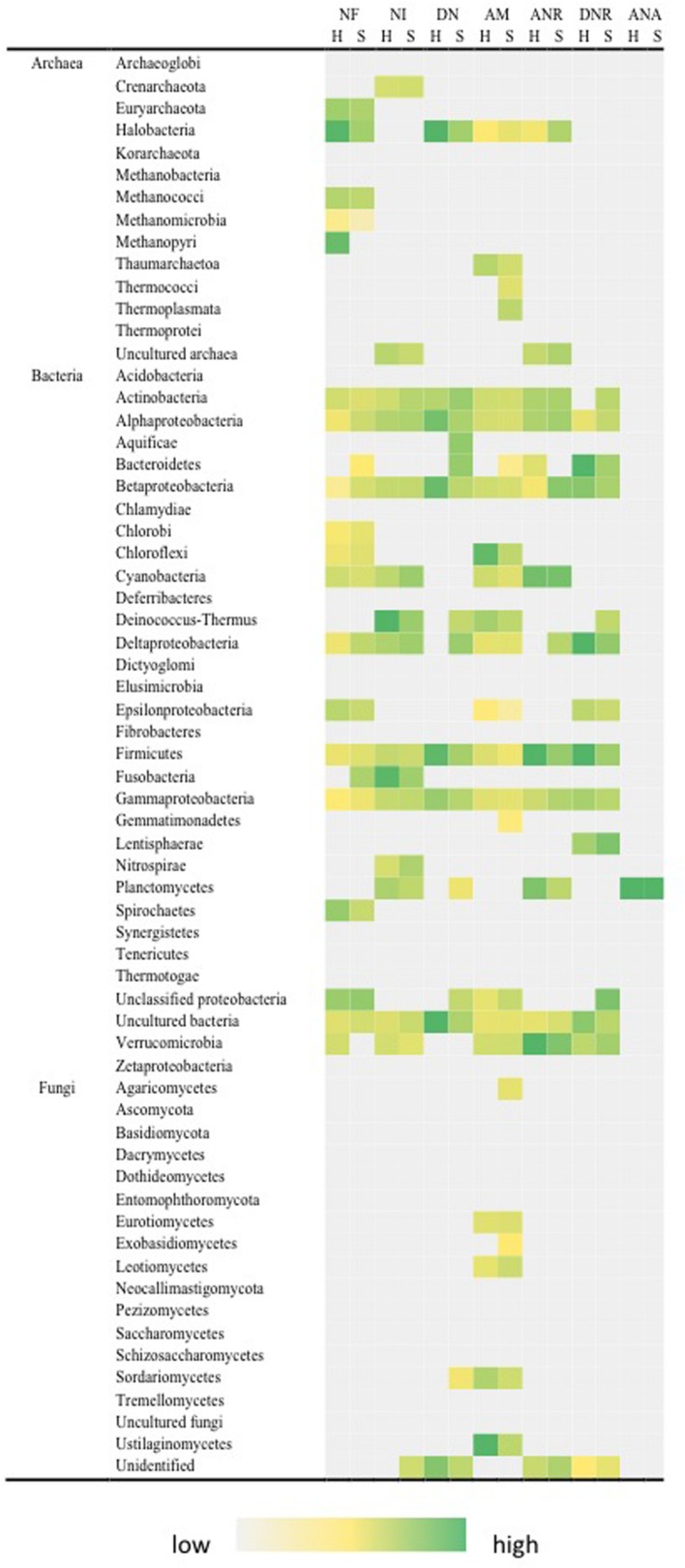
**Distribution of pathways for nitrogen transformation in soil (S) and hypolith (H) communities.** NF, nitrogen fixation; NI, nitrification; DN, dentrification; AM, ammonification; ANR, assimilatory nitrogen reduction; DNR, dissimilatory nitrogen reduction; ANA, anaerobic ammonium oxidation (Anammox). Color shading indicates relative hybridization intensity.

The key observation for nitrogen transformation was that the complete nitrogen cycle pathway was present in both hypolith and soils (**Figure 5**). Three phyla (Actinobacteria, Firmicutes, and Proteobacteria) possessed all pathways for the nitrogen cycle (excluding ANAMMOX) and these also reflected abundant rRNA-defined phyla in the system. Fixation of atmospheric nitrogen was an archaeal and bacterial pathway, with strongest signals from the phyla Halobacteria (Archaea), Methanopyri (Archaea) and Proteobacteria (Bacteria). Phyla that possessed nitrogenase genes for nitrogen fixation generally also possessed pathways for nitrification. The fungi generally displayed pathways related to ammonification. There was no significant difference between nitrogen fixation, nitrification and ammonification pathways between hypolith and soil communities. There were other significant differences between hypolith and soil communities. Soils supported significantly greater signal for denitrification (*F* = 111.639, *P* < 0.01), assimilatory nitrate reduction (*F* = 8.764, *P* < 0.05) and dissimilatory nitrate reduction (*F* = 28.246, *P* < 0.01). The ANAMMOX pathway was only present in Planctomycetes, and was a significantly greater in hypoliths (*F* = 10.369, *P* < 0.01).

### Stress Response Pathways

**FIGURE 6:**
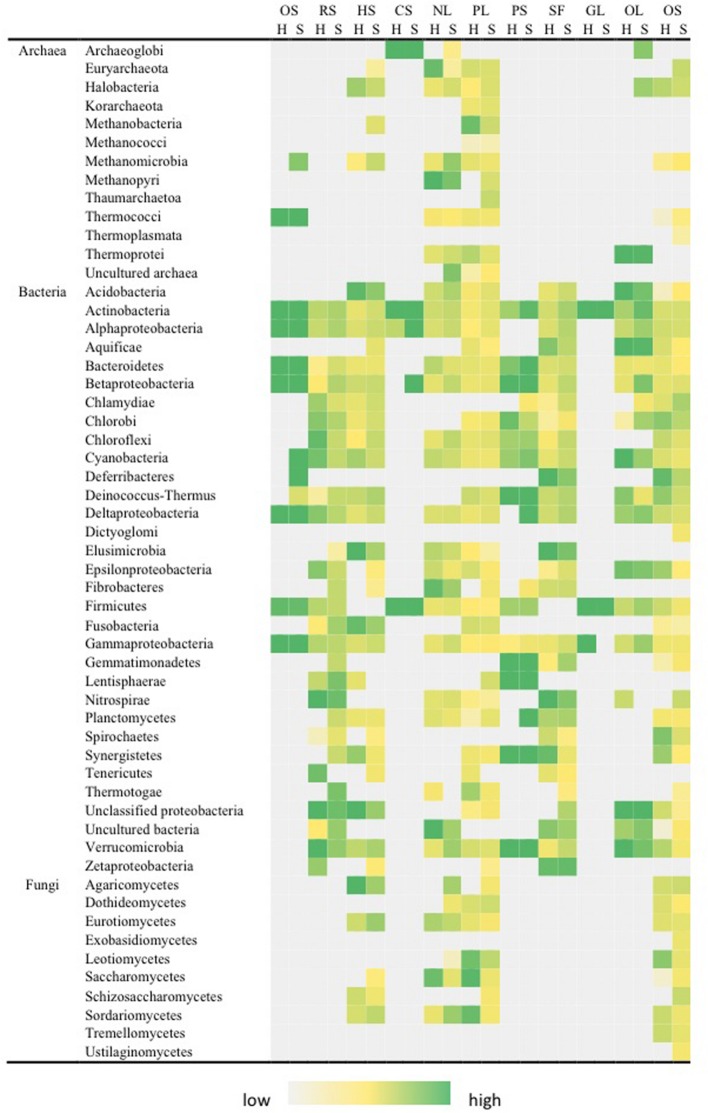
**Distribution of pathways for stress response in soil (S) and hypolith (H) communities.** OS, osmotic stress; RS, radiation stress; HS, heat shock; CS, cold shock; NL, nitrogen limitation; PL, phosphate limitation; PS, protein stress; SF, sigma factor; GL, glucose limitation; OL, oxygen limitation; OS, oxygen stress. Color shading indicates relative hybridization intensity.

Stress response pathways were the most variable among all functional pathways evaluated (**Figure 6**). The most abundant signals were related to nutrient and oxygen limitation, and these were present in archaea, bacteria and fungi. Other pathways related to protein and glucose limitation were only recovered for bacteria. Stress response pathways that are specifically related to Antarctic environments included osmotic, radiation (desiccation), heat and cold shock. Strongest signals for osmotic stress tolerance were recovered for Actinobacteria, Bacteroidetes, Cyanobacteria, Firmicutes, and Proteobacteria. Dessiccation-tolerance as evidenced by radiation stress response pathways was common to most bacterial phyla encountered in both niches. Heat shock was near-ubiquitous whilst cold shock pathways were detected only for Actinobacteria, Firmicutes, and Proteobacteria, plus the Archaeoglobi (Archaea). There were significant differences between hypolith and soil communities. Hypolith communities displayed greater signal for glucose (*F* = 22.374, *P* < 0.01) and phosphate (*F* = 109.952, *P* < 0.01) limitation, whilst in soils abiotic stress pathways including desiccation (*F* = 12.833, *P* < 0.05), heat shock (*F* = 254.23, *P* < 0.01) and oxygen stress (*F* = 12.855, *P* < 0.05) were more abundant.

## Discussion

This study provides a combined interrogation of taxonomic and functional diversity in Miers Valley, a long-term study site in the McMurdo Dry Valleys of Antarctica. The findings identify that soils are a reservoir of highly diverse bacterial taxa but that most of these are encountered with very low abundance. Hypolithic communities may be partially recruited from these soils and develop cyanobacteria-dominated biofilms with lower overall diversity than soils and with cyanobacteria comprising approximately half of all recoverable phylotypes. This is because the hypolithic niche provides microclimate conditions favorable to development of photosynthetic biofilms (Chan et al., 2012). These findings illustrate the potential connectivity between communities in these two niches, although the observation that soils adjacent to and beneath hypoliths are more similar to hypolith-free soils than hypolithic communities emphasizes the selective forces that operate on a highly localized level for these niches. The accumulation of extracellular polymeric compounds (EPS) and the development of intimate physical associations between microorganisms and mineral components in hypolithic habitats likely contribute to the formation of this specific selective microenvironment (de los Rios et al., 2014).

Our study contrasts with an earlier claim that Miers Valley hypolithic communities were dominated by proteobacteria (Makhalanyane et al., 2013). Other observations of Miers Valley hypoliths support the conclusions of our sequencing-based study that shows they are dominated by cyanobacteria (Cowan et al., 2010, 2012), as well as sequencing studies from other dry valleys locations using clone libraries (Wood et al., 2008; Pointing et al., 2009). Sequencing investigations on hypoliths in other environments also concur with our findings (Warren-Rhodes et al., 2006; Pointing et al., 2007; Wong et al., 2010; Lacap et al., 2011; Makhalanyane et al., 2012). Recent large-scale studies also indicate that hypoliths worldwide are typically dominated by cyanobacterial taxa (Bahl et al., 2011; Caruso et al., 2011).

It has been proposed that hypoliths in the Dry Valleys are wholly recruited from surrounding soils (Makhalanyane et al., 2013). Our study showed that just over a one third of hypolithic OTUs were shared with soil, but others were unique to hypoliths. Given that both studies had relatively low sequencing coverage and sample numbers, further research is needed to resolve this issue. Thus, current knowledge suggests that whilst the role of soil as a reservoir of bacterial diversity for hypoliths may apply in part (Makhalanyane et al., 2012), a proportion of hypolithic OTUs may also be recruited from elsewhere. It has been proposed that cyanobacterial recruitment to hypolithic communities in the Dry Valleys may occur in part due to aeolian dispersal from cyanobacterial mats originating in lakes (Wood et al., 2008; Pointing et al., 2009). Here, constraints on intra- and inter-valley dispersal are envisaged as a major driver of local biogeography (Pointing et al., 2015), and a recent study revealed most airborne microorganisms in Miers Valley were of local Antarctic origin rather than arriving via long-range dispersal (Bottos et al., 2013). The connectivity between soil and lithic communities in the Dry Valleys may emerge as complex. Not only are hypoliths likely dependent in part on soil for recruitment, but it has also been demonstrated that cyanobacteria from cryptoendolithic origin are also detectable in soil (Hopkins et al., 2009) and thus recruitment from rock to soil habitats may also occur. This is interesting in light of the recent discovery that hypolithic cyanobacteria can colonize micro-fractures and crevices in the substrate, thus displaying a chasmoendolithic growth habit (de los Rios et al., 2014).

The most abundant hypolithic cyanobacterial genera were *Microcoleus*, *Oscillatoria*, and *Synechococcus*. The oscillatorian cyanobacteria are common in hypolithic communities in cold and polar deserts (Wood et al., 2008; Pointing et al., 2009; Chan et al., 2012), although they are less common in hot desert hypoliths (Warren-Rhodes et al., 2006; Pointing et al., 2007; Bahl et al., 2011; Stomeo et al., 2013). It has been reasoned that this is because they display traits of an r-selected species in that they can grow rapidly during periods favorable to growth such as the polar summer, but are not well adapted to extreme aridity as dominant hot desert hypolithic cyanobacteria such as *Chroococcidiopsis* appear to be in a k-selection strategy (Pointing, 2016). *Microcoleus* is a keystone genus involved in formation of biological soil crusts (Belnap, 2003). However, this genus was not commonly encountered in 16S rRNA gene surveys of hypoliths in other locations (Chan et al., 2012; Pointing, 2016), and so this may indicate Miers Valley has a structured hypolith community with some similarity to the relatively more complex biological soil crusts (Belnap et al., 2003). A recent microscopy study demonstrated Miers Valley hypoliths can support a previously unappreciated high degree of internal organization (de los Rios et al., 2014). This is partly borne out by the finding that as with biological soil crusts, some Miers Valley hypoliths support diverse fungi and mosses as well as bacteria (Cowan et al., 2012). The genus *Synechococcus* is commonly associated with aquatic environments (Jing et al., 2005, 2009) and is not a dominant hypolithic taxon (Chan et al., 2012). Large areas of the valley floor hypolithic habitat in Miers Valley can become submerged during the brief Antarctic summer and so this may be reflected in the dominance of this taxon in our study compared to hypoliths in other environments. The most abundant heterotrophs were ubiquitous soil bacterial phyla from the Acidobacteria and Bacteroidetes, whilst phyla with known radiation (desiccation) tolerant genera such as *Deinococcus* and *Rubrobacter* were relatively under-represented compared to other studies of cold desert hypoliths (Wong et al., 2010). This suggests that adaptation to moisture stress may not be the principal driver of diversity in the Miers Valley system.

The functional ecology data obtained using the GeoChip microarray broadly matched known physiological traits for taxonomic assignments from sequence analysis, and this provided a strong ‘eco-triangulation’ for our dataset. Comparison to a previous study on a high inland valley location suggest Dry Valleys edaphic niches support communities with similar functional diversity (Chan et al., 2013), and a global soil metagenome comparison indicates Antarctic soils may be highly distinct from all other soils globally (Fierer et al., 2012). An analysis of functional traits for soil and hypolith communities revealed that the most abundant phyla also displayed greatest functional diversity. This metabolic plasticity may explain a central role for these abundant taxa as keystone species that perform the majority of ecological transformations and facilitate development of community richness (Chan et al., 2013). Many traits were shared across many phyla in both communities, and this indicates a level of functional redundancy at the community level that suggests communities may also be highly resilient, although the extent to which any resilience will confer an advantage in the abrupt changes expected for Antarctic systems is currently unclear. Our analysis revealed that bacteria were the most functionally diverse domain in soil and hypoliths. Coupled with the finding that they are the most abundant organisms by several orders of magnitude (Pointing et al., 2009), this suggests bacteria perform the majority of biogeochemical transformations.

Hypoliths clearly represent islands of enhanced potential for primary metabolic pathways, in particular for photosynthetic carbon fixation and carbohydrate metabolism. This is consistent with the notion that they occur as islands of productivity amid the generally depauperate landscape of desert soils (Pointing and Belnap, 2012; Pointing, 2016). In contrast, the relatively strong signals for catabolism of recalcitrant compounds in soils indicates that soil communities may have adapted to exploit these persistent, yet low energy, compounds in desert soils and this may be a feature throughout Dry Valleys soils (Chan et al., 2013). Nitrogen transformation potential appeared to be a widely distributed capability in both communities, and the lack of differentiation between communities in this regard might reflect the nitrogen limiting nature of the extreme oligotrophic Dry Valleys environment. The ability to fix nitrogen as evidenced by acetylene reduction has been demonstrated *in situ* for Antarctic hypolithic communities and this has been identified as a potentially important source of nitrogen input to the Dry Valleys ecosystem (Cowan et al., 2011). In terms of stress response pathways, soil communities were more adapted to abiotic stressors and this is consistent with the exposed nature of soils. Conversely adaptation to nutrient stress was more pronounced in hypoliths, suggesting that these more productive communities are also adapted to the fluctuating resource availability that can be envisaged since cyanobacteria are periodically active during ‘pulses’ of favorable growth conditions (Warren-Rhodes et al., 2007).

The study is not without limitations. For example the functional analysis using GeoChip microarray is not discovery-based and so novel adaptive traits are undetectable. Also the level of activity for the pathways detected remains unexplored, and measuring activity *in situ* should be a priority for future research. Nonetheless, this study has provided novel insight into the taxonomic and functional diversity of ice-free Antarctic soils in a system where ephemeral moisture sufficiency has resulted in hypolith and soil communities that differ markedly from those in the drier inland valleys. Whether these differences, such as the relatively high abundance of *Synechococcus* sp., actually translate to ecotypes with different levels of productivity for the system remains unknown. Overall, this study has identified highly diverse Dry Valleys communities dominated by actinobacteria in soil and cyanobacteria in hypoliths. Both communities displayed similar diversity-functionality relationships although beta diversity was higher in soils. The high level of functional redundancy suggests that some potential for resilience to change may occur within these communities, although further work to explicitly test this is required. The major differences in putative community functionality were that soils appeared relatively enriched in stress tolerance genes whereas hypoliths were enriched in genes related to tolerance of nutrient limitation. We conclude that these adaptations at the community level reflect the exposed nature of soil habitat compared to the ‘refuge’ habitat of quartz stones for hypolithic communities that may buffer climatic extremes but is a nutrient poor substrate. This may indicate that response to change could vary between soil and hypolith communities.

## Author Contributions

SP and DH conceived the study. AdR, DH, and SP conducted the field work. SW, DL-B, ML, SR, AdR, SA, JV, and JZ conducted experimental work. JC, CH, TC, AdR, and SP analyzed data. SP wrote the manuscript. All authors read and commented on the draft manuscript.

## Conflict of Interest Statement

The authors declare that the research was conducted in the absence of any commercial or financial relationships that could be construed as a potential conflict of interest.

## Footnotes

1 www.clontech.com

2 www.thermofisher.com

3 https://products.appliedbiosystems.com

4 https://www.r-project.org/

5 www.beckmancoulter.com

6 www.bmglabtech.com

7 www.454.com
