# Supplementary material for: Taxonomic and Functional Diversity of Soil and Hypolithic Microbial Communities in Miers Valley, McMurdo Dry Valleys, Antarctica
Source: Front Microbiol. 2016 Oct 20;7:1642. doi: 10.3389/fmicb.2016.01642 (PMC5071352; doi:10.3389/fmicb.2016.01642)

Taxonomic and functional diversity of soil and hypolithic microbial communities in Miers Valley, McMurdo Dry Valleys, Antarctica
Supplementary online material

Supplementary Figure S1. De-trended correspondence analysis was used to illustrate that t-RFLP defined taxa with greater distance from others in the two-dimensional plot explain more of the observed differences in diversity between the two substrates. Greatest contribution to between-community differences (i.e. soil vs. hypolith) was from Acidobacteria, Cyanobacteria and unidentified taxa. Note that significance testing is not possible with the DCA data.

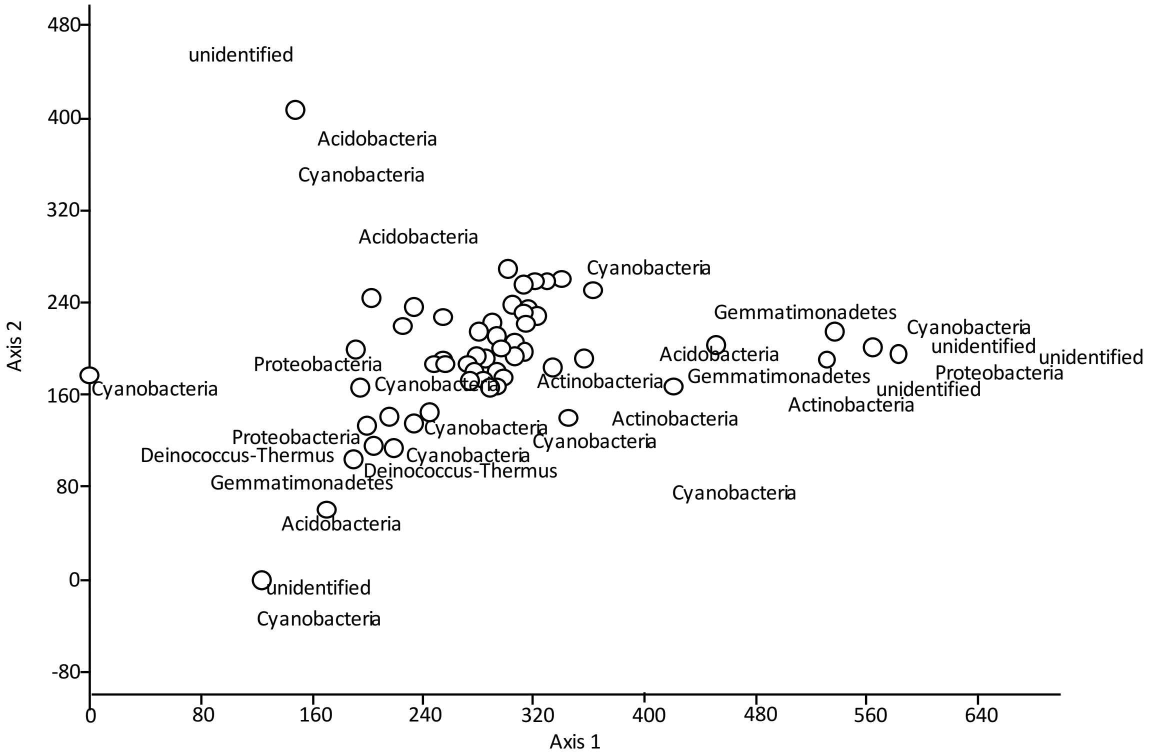

Supplementary Figure S2. Taxon identification for soil and hypolith bacteria using high throughput sequencing (Roche GS Junior 454). Taxonomic classification of 16S rRNA gene sequences was made using the ribosomal database project Classifier. The inner rings show phylum level identification and the outer rings show genus-level identification. Sequence data have been deposited in NCBI's sequence read archive under accession number SRA052054.1.

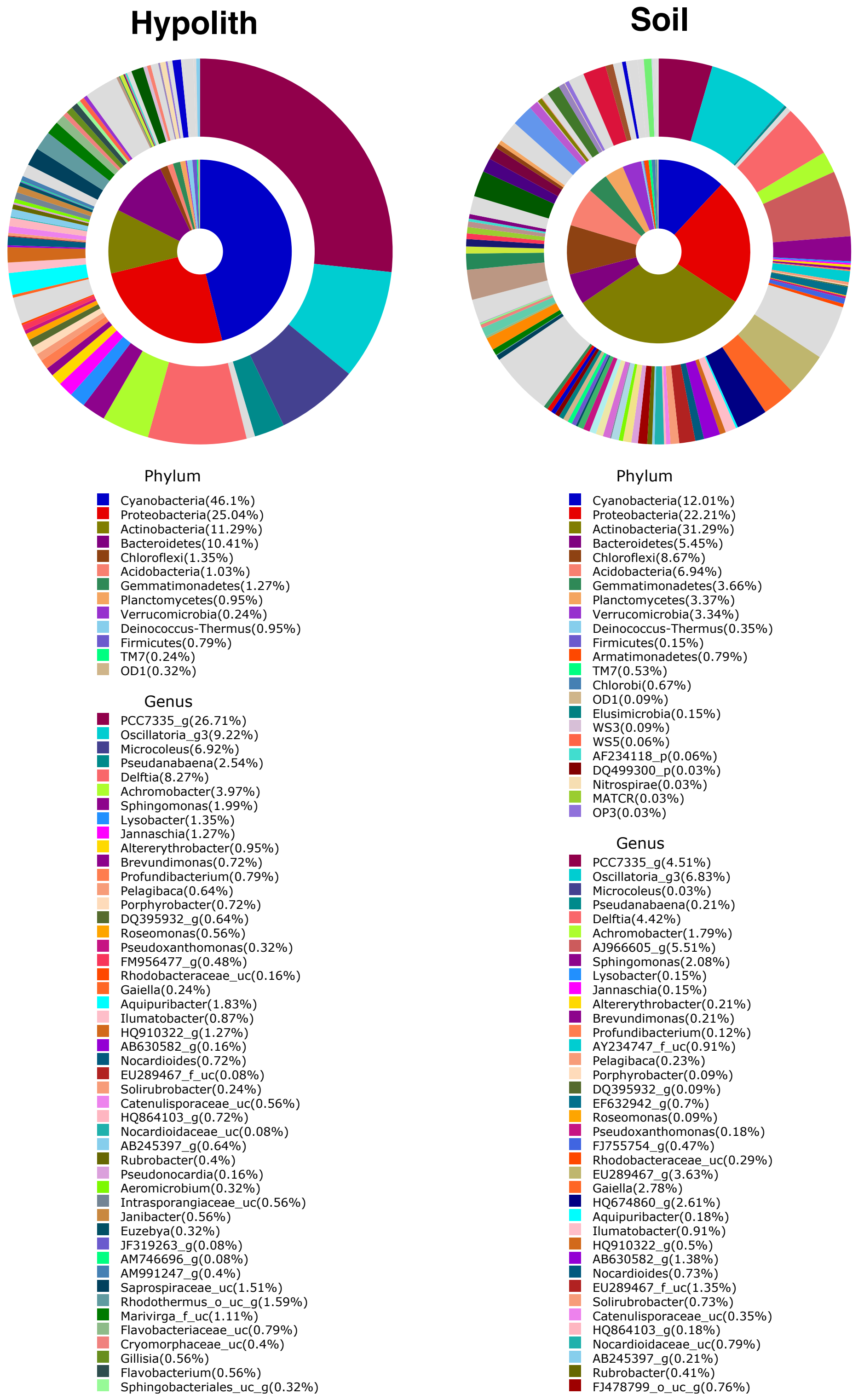

Supplement: Supplementary file 1 [file Image_1.PDF]
